# Organizational compassion and employee adversarial growth under various job control

**DOI:** 10.3389/fpsyg.2023.1294224

**Published:** 2023-12-20

**Authors:** Ting Nie, Xinqiang Zhao, Yanying Zheng

**Affiliations:** School of Business, Macau University of Science and Technology, Macau, China

**Keywords:** organizational compassion, work passion, self-worth, adversarial growth, job control

## Abstract

**Introduction:**

Adversity can bring stress and challenges to an individual's life, but many people who experience adversity also have positive changes. The formative mechanisms of individual adversarial growth have received widespread attention.

**Methods:**

A two-wave survey of 421 Chinese employees who experienced adversity during the COVID-19 epidemic was used to examine the influence mechanism of organizational compassion on adversarial growth and the moderating effect of job control.

**Results:**

Through correlation analysis, hierarchical regression, and bootstrap test on the cross-sectional data, the study has verified organizational compassion, work passion, self-worth, and adversarial growth form a chain mediating relation. Job control negatively moderates the indirect effect of organizational compassion on adversarial growth through work passion and self-worth, that is, the positive effect of organizational compassion on employee adversarial growth through work passion and self-worth is more pronounced under lower job control.

**Discussion:**

Organizational compassion can increase employee adversarial growth by enhancing their work passion and self-worth. Organizations should also pay more attention to those employees with lower job control who are in adversity, they are more likely to benefit from the organization's care and compassion.

## 1 Introduction

During the life course, individuals will inevitably experience various adversities and traumas, such as illness, divorce, layoffs, demotions, economic crises, epidemics, and natural disasters. In particular, over the past 3 years, many people have suffered a lot of negative impacts on their lives and work as a result of the COVID-19 epidemic (Dell'Osso et al., 2022). Some people will experience a range of negative effects and suffer from physical and psychological trauma. However, some people will gain positive changes from adversity by revisiting their lives and work to seek new opportunities and expand their social networks (Tedeschi et al., 2018). Personal adversarial growth has gained great attention in positive psychology, and many studies have been done on promoting positive changes and avoiding negative consequences when individuals experience adversities. Positive coping styles, family members, and peer support have all been shown to be effective predictors of individual adversarial growth (Sörensen et al., 2021; Knauer et al., 2022). However, most of the current research has focused on individual perspectives to explain adversarial growth, and research involving organizational perspectives is very limited.

As a social being, an individual will play multiple roles at the same time, and the impact of adversity will also be reflected in their various roles (Anglin et al., 2022). In particular, as one of the most important social roles, individual distress from adversity seriously affects interpersonal relationships and work performance in organizations, and can even generate deliberate sabotage and turnover behaviors (Worden, 2022). Therefore, it is not only the organization's corporate social responsibility to help employees cope with adversity and gain growth opportunities but also to enable the organization to achieve a stable and productive workforce. From a research perspective, exploring the influences of individual adversarial growth from an organizational aspect also can complement existing studies.

Individual compassion originates from empathy. But compassion does not only reflect a strong sense of empathy that individuals feel for others who are suffering but also emphasizes the desire to provide necessary support and help (Grant and Patil, 2012). Organizational compassion, on the other hand, is not limited to interactions between individuals but extends to collective actions. It occurs when an organization as a whole “notices,” “feels,” and “responds” to the suffering of its members (Kanov et al., 2004). Employees can benefit from the organization's compassion and care in times of adversity. Organizations can provide psychological guidance and necessary skills support to individuals facing work-related difficulties (major mistakes, demotions, pay cuts, etc.) by providing training, communication, demonstrations, etc. (Dodson and Heng, 2022). But more importantly, when individuals face difficulties in their personal lives (divorce, illness, loss of a loved one, large debts, etc.), organizations can also help them cope with these challenges through flexible work arrangements, financial assistance, two-way communication, low-interest or no-interest loans, legal assistance, etc. (Dodson and Heng, 2022). Emotional support and physical help provided by the organization enhance the bond and trust between the organization and its employees, which can significantly increase employee wellbeing (Oriol et al., 2023), and they are more likely to have higher enthusiasm and engagement in their work (Kanov et al., 2004; Dodson and Heng, 2022). According to role theory, social roles influence and permeate each other, and individuals will reduce their frustration due to the success of another role (Reiche et al., 2023). Thus, a positive work role also helps to increase an individual's self-worth, which is a subjective feeling about their own judgment (Covington, 1984). Based on social cognitive theory, individuals with higher self-worth are more confident in facing various adversities and actively searching for solutions and thus are more likely to acquire adversarial growth (Benight and Bandura, 2004; Yeung et al., 2023).

Many people perceive a loss of control over their lives because of the adversity they face, which may lead to a denial of self (Frazier and Caston, 2015; Mahmoudfakhe et al., 2023). If they can be given more autonomy on-time scheduling, workflow, task preference, etc., it can somehow help them regain confidence in their future. Moreover, Seeking control of the work environment is the core of workplace stress theory, which is considered to be the most important factor influencing expected occupational stress, happiness perception, and work involvement (Ahmad and Maochun, 2019; Huth and Chung-Yan, 2023). The effect of organizational compassion on employee adversarial growth should be more pronounced as a result of increased employee control over their work.

Based on a survey of Chinese employees who have experienced adversities during the COVID-19 outbreak, this study tries to examine the influence mechanism of organizational compassion on employee adversarial growth through work passion and self-worth, and to compare the impact differences at various job control levels. Finally, the practical implications of promoting employee adversarial growth through organizational compassion are discussed.

## 2 Theoretical basis and research hypotheses

### 2.1 Organizational compassion and adversarial growth

Compassion emphasizes that people are impressed by the suffering of others and would like to take action to help them (Atkins and Parker, 2012). People map and feel the pain of others, which is a form of psychological comfort given to reduce the pain of others (Ekman, 2007; Broadley et al., 2023). Compassion also exists in organizations and goes through the same three stages as individual compassion: “feeling pain—empathy—relief response” (Kanov et al., 2004). Organizational compassion occurs when members of the organization collectively notice, feel, and respond to the pain experienced by other members in the system (Dodson and Heng, 2022). Noticing involves being aware of other people's suffering. Feeling involves empathizing, or sympathizing with other person's hurt, pain, or worry. Responding compassionately involves taking actions to alleviate or eliminate other person's pain (Frost et al., 2000). Different from individual compassion, the process of organizational compassion operates in a collective manner and involves three key elements: legitimation, propagation, and coordination (Dodson and Heng, 2022). This is demonstrated by granting individuals the freedom to express their feelings and actions in specific ways in the organization (Kanov et al., 2017), promoting collective attention through information dissemination among organization members, empathizing with others through leadership modeling (Dutton et al., 2014), and helping members to cope with suffering through organizational practices or policies such as employee assistance programs or voluntary donation (Frost et al., 2006). Compassion in organizations emphasizes how the organizational environment influences individual ability and intention to show compassion, not only as individuals to care for others but also as organizational members to help them (Dutton et al., 2014). Organizational values, culture, systems, and structure can have a significant impact on organizational compassion, which reflects the positive rewards that organizations give to their employees (Kanov et al., 2017). When an organization's culture values pain expression and encourages emotion sharing, it is easier for organization members to empathize with the pain of others (Dodson and Heng, 2022). In a tolerant organizational climate, compassion as an organizational value is more conducive to pro-social responses from its members (Fehr and Gelfand, 2012). Organizational leaders can also enhance organizational compassion by modeling it through a broader emotional expression and by showing high concern for employees (Kroth and Keeler, 2009; Lanaj et al., 2022). It can facilitate the establishment of a harmonious communication relationship between the leaders and their subordinates (Floyd, 2016). Organizational compassion can also contribute to the wellbeing of employees by reducing their perceived stress and increasing their work engagement (Eldor, 2018; Cheng et al., 2023; Paganin et al., 2023).

Individuals would re-evaluate and establish their new value after experiencing trauma such as severe diseases, accidents, or emergencies, and improve their adaptability so as to gain post-traumatic growth. It might take place in self-knowledge, interpersonal communication, attitude toward life, and so on (Tedeschi and Calhoun, 2004; Tedeschi, 2020). Joseph et al. (2008) pointed out that positive changes also may occur when individuals face different kinds of adversity. Adversarial growth reflects changes in the individual's assumptions about self and the world in response to adversity. It represents a normative emotional-cognitive process that promotes positive personality development as well as enhanced resilience and the adaptive function (Joseph, 2021). The challenge of core beliefs in adversity is the prerequisite for adversarial growth. Once their core beliefs are disputed, the individual's psychological imbalance will rebuild a new core belief system (Tedeschi, 2020). In this process, individuals would re-examine and re-recognize knowledge and assumptions about themselves, others, and the world, thereby promoting the occurrence of adversarial growth (Connerty and Knott, 2013; Colbert and Willmot, 2021). Therefore, cognitive reappraisal is considered the most effective strategy to deal with adverse events. It can help individuals regain control and start proactive responses (Cárdenas Castro et al., 2019). In line with Social Cognitive Theory, individuals form perceptions of others or things based on social information in the environment, which influences their responses as well as their behaviors (Benight and Bandura, 2004). When the organization's culture values the expression of suffering and emphasizes sharing emotions with those who are in adversity, then members are more likely to empathize with others' pain and provide the necessary support (Kanov et al., 2017). The warmth and responsiveness that employees receive from the organization and their colleagues can help individuals recover from negative emotions, re-evaluate their perceptions, and begin self-improvement (Kanov et al., 2017; Joseph, 2021). Meanwhile, the support and help from the organization and colleagues provide the basis for individuals to take action, facilitate their coping with difficulties, and gain opportunities for growth (Joseph, 2021). Therefore, research hypothesis 1 is proposed.

*H1: Organizational compassion has a positive impact on individual adversarial growth*.

### 2.2 The mediating role of work passion

Work passion is an individual's continuous, positive, meaningful and happy state (Zigarmi et al., 2009; Pollack et al., 2020). It comes from cognitive and emotional assessments of various work or organizational situations, which can lead to constructive work intentions and behaviors. Vallerand et al. (2003) defined work passion as the value and meaning that an individual experience at work, emphasizing a sincere love of work and a willingness to invest time and energy in it. They proposed that work passion can be divided into two types: harmonious passion (HP) and obsessive passion (OP); Obsessive passion refers to a controlled internalization in one's identity that can create internal pressure to engage in the activity. Harmonious passion refers to an autonomous internalization that leads individuals to engage in the activity they like (Landay et al., 2022). Cooperation among employees, fair evaluation within the organization, and open and transparent procedures are all conducive to employee work passion (Zigarmi et al., 2011; Breu and Yasseri, 2023). Career development can enhance employees' intrinsic motivation, increase work interest, and promote work passion. The study by Dubreuil et al. (2014) found that work passion can positively predict work involvement and job performance. Employees with high work passion have more fulfilling work and life with higher satisfaction (Chummar et al., 2019). At the same time, they are more willing to improve their cognitive ability to explore new knowledge and realize their self-worth (De Clercq et al., 2013). Organizational compassion can reduce negative social comparisons, increase the empathy level of organizational members, and contribute to a positive emotional climate within the organization. Individuals may face adversity in different areas. The reassurance of managers, the assistance of coworkers, and the application of management practices can be effective in helping individuals resolve professional adversities and stimulate a harmonious passion for work (Kanov et al., 2017). According to Social Role Theory, individuals play multiple roles simultaneously, and each role interpenetrates the other (Anglin et al., 2022). When experiencing personal adversities, the individual desires to alleviate anxiety through the accomplishments of other social roles, especially obtaining recognition at work (Anglin et al., 2022). The empathy of managers, the listening of colleagues, and flexible arrangements can simultaneously inspire individuals to have obsessive passion and harmonious passion for their work (Dutton et al., 2014; Kanov et al., 2017). They try to prove themselves, regain self-confidence, and reward the support from the organization and their peers (Settoon et al., 1996; Joseph, 2021). Increased work passion and confidence in the workplace will make individuals more likely to actively seek out opportunities to learn and grow. Therefore, we propose research hypothesis 2:

*H2: Organizational compassion can influence individual adversarial growth through work passion*

### 2.3 The mediating role of self-worth

Xiting Huang (1998) defined self-worth as the positive emotional experience of oneself generated in the process of self-recognition and evaluation in social life. It is durable and developed into a relatively stable trait. Individuals like and accept themselves due to their own values, and self-worth is a subjective feeling about their own judgment (Covington, 1984). Therefore, self-worth is also considered to be the gap between the true self and the ideal self. The larger the gap is, the lower self-worth is (Burwell and Shirk, 2006; Aghatabay et al., 2023). Social recognition, perceived support, learning ability, parenting style, interpersonal relationship, family economic status, religious belief, and personal traits, all have significant impacts on the formation and development of self-worth, which in turn affect individual mental health and behaviors (Corr, 1996; Kusina and Exline, 2021). Self-worth can predict the individual subjective wellbeing and effort. Individuals with low self-worth also typically have low subjective wellbeing and their future achievements may be affected (Furnham and Cheng, 2000; Fairlamb, 2022). In general, the importance of a domain to an individual affects the individual's self-evaluation in that domain (Rosenberg et al., 1995). Fairlamb (2022) pointed out that work is of great value to individuals. Therefore, the work role will be one of the effective ways for individuals to gain self-worth. Social Cognitive Theory states that human activities are determined by the interaction of individual behavior, individual cognition, and the external living environment. An individual's experience in the environment influences his or her perception of others and of the self (Benight and Bandura, 2004). The norms and climate of an organization or work team can impact the willingness of members to share their personal situations and painful feelings with others (Madden et al., 2012; Kanov et al., 2017). When leaders themselves show compassion and model appropriate ways of responding, it is legitimized as a valued and worthwhile behavior that encourages other organization members to respond to suffering as well (Lilius et al., 2011; Dodson and Heng, 2022). Especially for individuals in adversity, empathy and care from the organization and colleagues can give them warmth and help and restore their confidence (Eldor, 2018). Compassion within the organization also influences whether individuals in adversity have enough trust to confide with others, which is a start to face reality and find solutions (Dutton et al., 2014). As a return to the organization, individuals will be more enthusiastic in their work, strive to realize their self-worth, and obtain opportunities for growth even in adversity (Mruk, 2013; Colbert and Willmot, 2021). Therefore, hypotheses 3 and 4 are proposed:

*H3: Organizational compassion can influence individual adversarial growth through self-worth*.

*H4: Organizational compassion can influence individual adversarial growth through work passion and self-worth*.

### 2.4 The moderating role of job control

Job control is defined as the ability to make decisions about work, which emphasizes that individuals can creatively use new skills or facilitate career development (Karasek, 1979). When job demands are elevated, organizations typically simultaneously increase employee job control to promote psychological wellbeing, work enthusiasm, and commitment. Job control describes the degree of employee autonomy in the workplace (Turksoy and Tutuncu, 2021). They can determine the order and manner in which tasks are completed and identify individual responsibilities and job content (Huth and Chung-Yan, 2023). When employees are allowed to intervene and change the work process, it can reduce their negative reactions due to the lack of resources in the environment (Hobfoll, 2001; Ibrahim et al., 2019; Jung et al., 2023). Also, job control can enhance individual perceptions of wellbeing and job satisfaction (Fila et al., 2017; Ahmad and Maochun, 2019). The study of Wielenga-Meijer et al. (2010) found that job control has a significant correlation with learning motivation. In the case of higher work intensity, employees with higher job control are more innovative at work (Dediu et al., 2018). In general, jobs with low work demands and high control opportunities generate less stress (Theorell et al., 1990; Jung et al., 2023). Although they may be affected by a lack of challenges, they still have opportunities for development. Individuals in adversity are prone to perceive a loss of control and self-denial, and the comfort and support from managers and co-workers help to meet their basic psychological needs and enhance hope for the future (Bammens, 2016). Especially for employees who have low autonomy in their work, they may not get high emotional rewards directly from the work itself. When they face adversity, the value of organizational compassion becomes even more evident. Organizations can inspire their potential by encouraging them to set achievable goals and develop realistic plans. They are more likely to achieve adversarial growth due to a supportive organizational environment (Colbert and Willmot, 2021). The positive effect of organizational compassion on adversity growth through work passion and self-worth should be more pronounced when individuals have a low level of job control. Therefore, hypotheses 5 and 6 are proposed:

*Hypothesis 5: Job control negatively moderates the indirect impact of organizational compassion on individual adversarial growth through work passion*.

**Figure 1:**
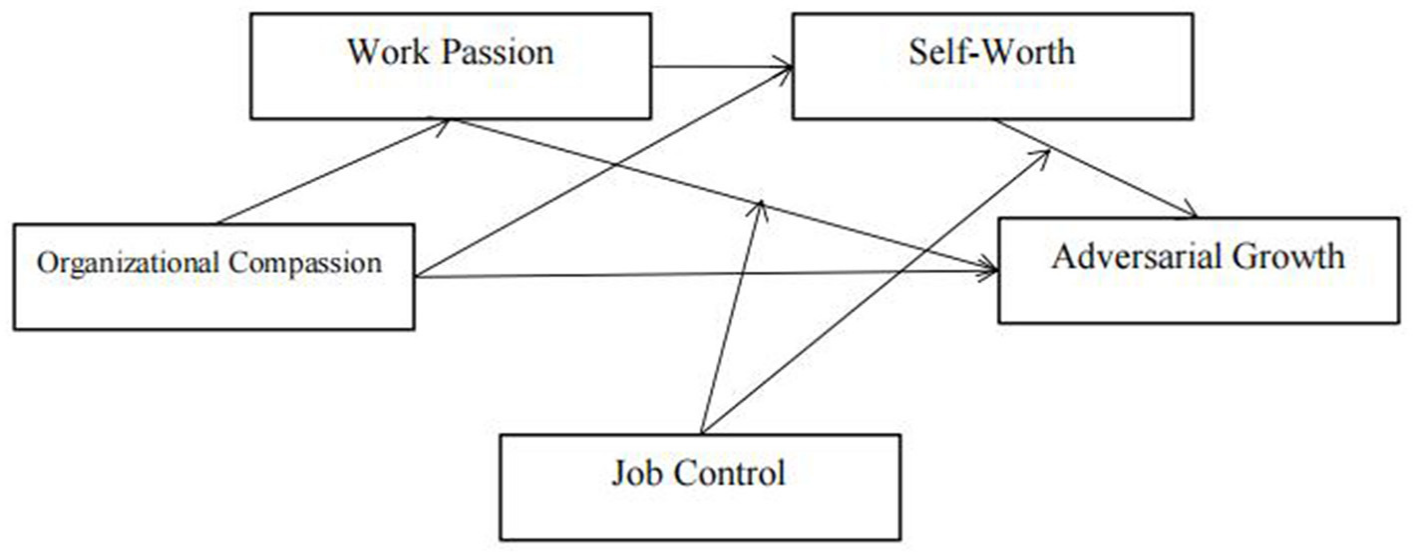
Theoretical framework.

*Hypothesis 6: Job control negatively moderates the indirect impact of organizational compassion on individual adversarial growth through self-worth*. The theoretical framework of the study is presented in Figure 1.

## 3 Method

### 3.1 Sample and data collection

This study examines the mechanism of organizational compassion on employees' adversarial growth and the moderating effect of job control by investigating employees' perceptions after experiencing adversity. Adversity was defined with reference to the major life events mentioned in the Social Readjustment Rating Scale (Holmes and Rahe, 1967). In our study, ten negative events were identified as adversities: widowhood, divorce, separation, incarceration, death of a family member, unemployment, sexual disorders, major illness, substantial debt, and demotion. Respondents were first asked if they had experienced the ten adversities that had a significant impact on their lives. Respondents who answered negatively terminated the survey, while those who answered positively continued to complete the questionnaire based on their personal experiences. Data were from companies in Guangdong, Heilongjiang, Jilin, Inner Mongolia, Liaoning, Ningxia, and other provinces and cities of China. In order to reduce the effect of common method deviation, the data were collected in two time periods with one-month interval. T1: Data on organizational compassion, self-worth, and job control were collected by convenience sampling. T2: Data on work passion, adversarial growth, and demographic characteristics were collected by matching tele numbers. Siz hundred questionnaires were distributed, and 421 questionnaires were recovered with a recovery rate of 70.2%.

The study was reviewed by the Research Ethics Committee of Business School at Macau University of Science and Technology. All methods in the study were performed in accordance with the Declaration of Helsinki. Respondents were informed of the study's purpose before the survey. Data were guaranteed to be used for academic research only. Respondents completed the questionnaire voluntarily and were free to terminate their responses at any time. Incomplete questionnaires were considered invalid.

In the valid sample, there are 218 male respondents (51.9%), and 203 female respondents (48.2%). The ratio of men to women is basically balanced. In terms of age distribution, there are 142 respondents from the 20–30 age group (34%), 192 respondents from the 31–40 age group (45.6%), 78 respondents from the 41–50 group (18.5%) and eight respondents are more than 50 years old (1.9%). In terms of education level, most of the respondents have received high education, 328 respondents have a bachelor's degree or higher. Respondents are mostly knowledge workers. 258 (58.9%) of the respondents are frontline staff and 173 (51.1%) are junior managers; middle and senior managers were not included in this study.

### 3.2 Measurement

Likert five-point scales were used to measure the relevant variables (from completely disagree to completely agree).

**Organizational compassion** exists when organization members collectively notice, feel, and respond to the pain experienced by other members (Kanov et al., 2004). Organizational compassion was measured with the scale developed by Lilius et al. (2008), and its internal consistency coefficient is 0.864. Respondents' perceived organizational compassion is relatively high (Mean = 3.86; SD = 0.500).

**Work passion** is the value that an individual could experience at work, and is willing to invest time and energy in this work (Vallerand et al., 2003). Work passion was measured with the scale developed by Vallerand et al. (2003), and its internal consistency coefficient is 0.880. Respondents' work passion is at a medium to high level (Mean = 3.81; SD = 0.599).

**Self-worth** is the positive emotional experience of oneself generated in the process of self-recognition and evaluation in social life (Xiting Huang, 1998). Self-worth was measured with the scale developed by Xiting Huang (1998), and its internal consistency coefficient is 0.815. Respondents' self-worth is at a medium to high level (Mean = 3.74; SD = 0.577).

**Adversarial growth** is the psychological wellbeing and changes in assumptions about the self and the world in the process of coping with adversity (Joseph et al., 2008). Adversarial growth was measured with the scale developed by Cann et al. (2010), and its internal consistency coefficient is 0.953. Respondents' perceived adversarial growth is at a medium to high level (Mean = 3.79; SD = 0.598).

**Job control** is the autonomy or decision-making authority of employees at work (Ganster and Fusilier, 1989). Job control was measured with the scale developed by Gonzalez-Mulé and Cockburn (2017), and its internal consistency coefficient is 0.916. Respondents' job control is relatively high (Mean = 3.83; SD = 0.681).

### 3.3 Analytical approach

Spss 26, Process 3.4, and Amos 26 software were used to analyze the data and validate the study hypotheses. Firstly, we conducted confirmatory factor analyses (CFAs) using Amos 24 to test the measurement model and the common method bias in the study. Descriptive statistics and correlation analysis were then used to explore the levels and preliminary relationships of the variables. Hierarchical Regression was conducted using Spss 26 to examine the direct effect of organizational compassion on individual adversarial growth, as well as the mediating effect of work passion and self-worth. Finally, Process 3.4 was used to test the moderating effect of the work control. We also compared the boundary differences between high (Mean + 1 SD) and low (Mean − 1 SD) job control for the indirect effect of organizational compassion on individual adversity growth.

## 4 Results

### 4.1 Confirmatory factor analysis

First of all, Confirmatory Factor Analysis was conducted. The model fit of the five-factor model (organizational compassion, work passion, self-worth, adversarial growth, job control) is significantly better than other alternative models (χ^2^/*df* = 1.703, NFI = 0.936, TLI = 0.968, CFI = 0.972, RMSEA = 0.041, RMR = 0.020), which has reached an acceptable level. The model fit of the single-factor model is far from acceptable (χ^2^/*df* = 6.875, NFI = 0.731, TLI = 0.728, CFI = 0.759, RMSEA = 0.118, RMR = 0.048). Therefore, the common method bias is not serious in the study.

### 4.2 Correlation statistics

The results of correlation statistics are shown in Table 1. Gender, age, and education are considered as control variables, and all the variables in the study have significant correlations. (1) Organizational compassion is significantly positively correlated with work passion (*r* = 0.665, *p* < 0.01), self-worth (*r* = 0.479, *p* < 0.01), and adversarial growth (*r* = 0.694, *p* < 0.01). (2) Work passion is significantly positively correlated with self-worth (*r* = 0.570, *p* < 0.01) and adversarial growth (*r* = 0.774, *p* < 0.01). (3) Self-worth is significantly positively correlated with adversarial growth (*r* = 0.568, *p* < 0.01). (4) Job control is significantly positively correlated with organizational compassion(*r* = 0.458, *p* < 0.01), work passion (*r* = 0.696, *p* < 0.01), self-worth (*r* = 0.537, *p* < 0.01), and adversarial growth (*r* = 0.622, *p* < 0.01).

**Table 1:** Mean, standard deviation, and correlation statistics (*n* = 421).

	**Mean**	**SD**	**1**	**2**	**3**	**4**	**5**	**6**	**7**	**8**
1.Gender	1.48	0.50	–							
2.Age	2.88	0.77	0.07	–						
3.Edu	1.88	0.59	−0.06	−0.17^**^	–					
4.OC	3.86	0.50	−0.01	0.08	0.08	**(0.70)**				
5.WP	3.81	0.60	0.04	0.22^**^	−0.01	0.67^**^	**(0.85)**			
6.SW	3.74	0.58	0.08	0.10^*^	0.11^*^	0.48^**^	0.57^**^	**(0.74)**		
7.AG	3.79	0.60	0.04	0.11^*^	0.02	0.69^**^	0.77^**^	0.57^**^	**(0.82)**	
8.JC	3.83	0.68	0.07	0.18^**^	0.08	0.46^**^	0.70 ^**^	0.54^**^	0.62^**^	**(0.77)**

^**^p < 0.01, ^*^p < 0.05; OC, organizational compassion; WP, work passion; SW, self-worth; AG, adversarial growth; JC, job control. The bold value on the diagonal is the AVE root value of each variable.

### 4.3 Hypothesis testing

Hypothesis 1 proposed that organizational compassion is positively related to adversarial growth. In Table 2, results indicated that when controlling gender, age, and education, organizational compassion has a significant positive effect on adversarial growth (β = 0.691, *p* < 0.001), and hypothesis 1 is supported.

**Table 2:** Hierarchical regression of mediating effect (*n* = 421).

**Regression equation**			**Regression coefficients**					**95% Confidence interval**	
	**DV**	**IV**	*R* ^2^	***F***	β	***t***	***p***	**Lower**	**Upper**
M1	AG	Gender	0.49	98.09	0.03	0.90	0.37	−0.05	0.12
		Age			0.05	1.31	0.19	−0.02	0.09
		Edu			−0.02	−0.57	0.57	−0.09	0.05
		OC			0.69	19.51	0.00	0.74	0.91
M2	WP	Gender	0.47	92.73	0.03	0.77	0.44	−0.05	0.12
		Age			0.16	4.49	0.00	0.107	0.18
		Edu			−0.01	−0.21	0.84	−0.08	0.07
		OC			0.65	18.18	0.00	0.70	0.87
M3	AG	Gender	0.66	160.05	0.02	0.55	0.58	−0.05	0.09
		Age			−0.05	−1.55	0.12	−0.08	0.01
		Edu			−0.02	−0.55	0.59	−0.07	0.04
		OC			0.57	14.51	0.00	0.49	0.65
		WP			0.32	8.20	0.00	0.29	0.47
M4	SW	Gender	0.25	34.07	0.08	1.76	0.08	−0.10	0.18
		Age			0.08	1.80	0.07	−0.01	0.12
		Edu			0.09	2.12	0.04	0.01	0.17
		OC			0.47	10.84	0.00	0.44	0.63
M5	AG	Gender	0.56	104.19	0.01	0.26	0.80	−0.07	0.09
		Age			0.02	0.69	0.49	−0.03	0.07
		Edu			−0.05	−1.45	0.15	−0.12	0.02
		OC			0.55	14.70	0.00	0.57	0.74
		SW			0.31	8.16	0.00	0.24	0.40

OC, organizational compassion; WP, work passion; SW, self-worth; AG, adversarial growth.

Hypotheses 2 and 3 proposed that work passion and self-worth mediates the relationship between organizational compassion and adversarial growth. Three-step regression was used to test the mediating effect of work passion and self-worth with the approach suggested by Baron and Kenny (1986). In Table 2, results of Models 1, 2, and 3 indicated that when controlling gender, age, and education, work passion has a partial mediating effect between organizational compassion and adversarial growth (*R*^2^ = 0.66, *F* = 160.05). Hypothesis 2 is verified. Results of Models 1, 4, and 5 indicated that when controlling gender, age, and education, self-worth has a partial mediating effect between organizational compassion and adversarial growth (*R*^2^ = 0.56, *F* = 104.19). Hypothesis 3 has been verified.

Hypothesis 4 proposed that organizational compassion, work passion, self-worth, and adversarial growth form a chain mediating relation. In Table 3, the direct and indirect effects of organizational compassion on adversarial growth are tested with the approach suggested by Hayes (2017). The chain mediation effect of work passion and self-worth between organizational compassion and adversarial growth is significant [*b* = 0.05, SE = 0.02, 95% CI (0.03, 0.08)]. Hypothesis 4 is supported.

**Table 3:** Chain mediation analysis (*n* = 421).

	**Effect**	**SE**	**LLCI**	**ULCI**
Total direct effect	0.35	0.05	0.26	0.44
Total indirect effect	0.48	0.05	0.39	0.55
**Indirect effect**				
Path 1: OC - WP - AG	0.40	0.43	0.31	0.48
Path 2: OC - SW - AG	0.31	0.02	0.01	0.07
Path 3: OC - WP - SW - AG	0.05	0.02	0.03	0.08

OC, organizational compassion; WP, work passion; SW, self-worth; AG, adversarial growth; LLCI, lower level of 95% confidence interval; ULCI, upper level of 95% confidence interval.

Hypothesis 5 proposed that Job control moderates the indirect impact of organizational compassion on adversarial growth through work passion, such that the relationship is stronger when employee job control is lower (vs. high). Results in Table 4 show that the moderating effect of Job control is significant [*b* = −0.05, SE = 0.03, 95% CI (−0.14, −0.01)]. As expected: the indirect influence of organizational compassion on adversarial growth through work passion is stronger when employee job control is low [−1 SD; *b* = 0.38, SE = 0.05, 95% CI (0.29, 0.45)] in comparison with the association when job control is high [+1 SD; *b* = 0.31, SE = 0.05, 95% CI (0.20, 0.41)]. Hypothesis 5 is supported.

**Table 4:** Moderating effect and moderated mediating effect (*n* = 421).

	**Moderating effect**						**Moderated mediating effect**				
**Variables**		**Effect**	**SE**	***p***	**LLCI**	**ULCI**		**Index**	**SE**	**LLCI**	**ULCI**
WP	Int	−0.06	0.03	0.03	−0.12	−0.01		−0.05	0.03	−0.14	−0.01
	L	0.48	0.05	0.00	0.38	0.58	L	0.38	0.05	0.29	0.49
	H	0.39	0.05	0.00	0.29	0.50	H	0.31	0.05	0.20	0.41
SW	Int	−0.08	0.04	0.03	−0.16	−0.01		−0.05	0.03	−0.10	−0.01
	L	0.23	0.05	0.00	0.14	0.32	L	0.13	0.03	0.08	0.19
	H	0.12	0.01	0.01	0.02	0.22	H	0.07	0.03	0.01	0.13

WP, work passion; SW, self-worth; LLCI, lower level of 95% confidence interval; ULCI, upper Level of 95% confidence interval.

Hypothesis 6 proposed that Job control moderates the indirect impact of organizational compassion on adversarial growth through self-worth, such that the relationship is stronger when employee job control is lower (vs. high). Results in Table 4 show that the moderating effect of Job control is significant [*b* = −0.05, SE = 0.03, 95% CI (−0.10, −0.01)]. As expected: the indirect influence of organizational compassion on adversarial growth through self-worth is stronger when employee job control is low [−1 SD; *b* = 0.13, SE = 0.03, 95% CI (0.08, 0.19)] in comparison with the association when job control is high [+1 SD; *b* = 0.07, SE = 0.03, 95% CI (0.01, 0.13)]. Hypothesis 6 is supported.

## 5 Discussions and conclusions

Although adversity can bring great challenges and stress to an individual's life and work, many people will also make positive changes when coping with adversity (Tedeschi, 2020). Through the two-wave survey on Chinese employees who experienced adversity during the COVID-19 epidemic, this study examines the influence mechanism of organizational compassion on adversarial growth and the moderating effect of job control. The finding indicates that work passion and self-worth have moderating effects between organizational compassion and individuals' adversarial growth, which means that organizational compassion can enhance individuals' adversarial growth through work passion and self-worth. Job control moderates the indirect effect of organizational compassion on adversarial growth. For individuals with low job control at the workplace, the indirect impact of organizational compassion on adversarial growth through work passion and self-worth is stronger. All hypotheses in this study are supported.

### 5.1 Theoretical implications

Previous studies have mostly explained antecedents and consequences of adversarial growth from the individual perspective (Joseph, 2021; Yeung et al., 2023), and relatively lacking is the exploration of organizational roles when individuals face adversity. This study explores the role of organizational compassion in the formation of individual adversarial growth from an organizational perspective. The findings indicate that organizational compassion can promote adversarial growth by increasing an individual's work passion and self-worth. This result reflects the reciprocal relationship between the organization and employees in Social Exchange Theory. For employees in adversity, the empathy and supportive measures of the organization can enable them to experience passion in their work. This not only enhances the individual's sense of self-worth but also helps the individual regain confidence to cope with adversity. Meanwhile, the conclusions of this study are also consistent with previous research on personal compassion. When people receive love and compassion from family and friends, they feel warmth and gain hope, which significantly predicts the occurrence of adversarial growth (Yeung et al., 2022). The work role and the family role, as the two most important roles, are mutually reinforcing and supportive (Reiche et al., 2023). Individuals may benefit from organizational compassion when they are faced with either professional or personal life adversity (Kanov et al., 2017). The organization as a whole acknowledges and empathizes with the challenges faced by its members (Kanov et al., 2017). By offering emotional and policy assistance, such as flexible work arrangements, empathetic managers, understanding coworkers, and access to employee assistance programs, the organization can help individuals to effectively navigate adversity and facilitate personal growth. This further confirms the main points of Social Cognitive Theory, employees will transfer these positive feelings from the work environment into cognitive improvements (Benight and Bandura, 2004). They will show more passion in their work and continue to improve their self-worth. Individuals with higher self-worth are more confident in facing various adversities and actively searching for solutions and thus are more likely to acquire adversarial growth (Eldor, 2018).

Job control was validated as a boundary condition for the relationship between organizational compassion and individual adversarial growth. The positive effect of organizational compassion on employee adversarial growth through work passion and self-worth is more pronounced when the employee has lower job control. This is significantly different from many previous studies that have emphasized the positive effects of job control such as increased job engagement, high work commitment, low stress, and more innovative behaviors (Dediu et al., 2018; Becker et al., 2022). The findings of this study show that in the case of lower work authorization, employees are more likely to gain adversarial growth due to organizational compassion. Empowerment gives employees control over their work and reduces their sense of loss due to adversity (Sawatsky et al., 2020). Individuals with low job control exacerbate the helplessness associated with adversity relative to individuals with high job control. They may prefer understanding and recognition from their supervisors and also from other organization members. Thus, the positive effect of organizational compassion is more pronounced in employees with lower job control. They are more likely to derive strength from the empathy and supportive policies of the organization, which enhances their enthusiasm for their work, as well as their self-evaluation (Eldor, 2018). As Self-determination Theory suggests: autonomy and control satisfy an individual's need for behavioral choices (Ryan et al., 2022). External support plays a more important role when this need is not met. Relative to individuals with high job control, emotional compassion and care are more likely to motivate individuals with low job control to face reality, find solutions, and gain opportunities to grow from adversity (Kanov et al., 2017; Joseph, 2021). Therefore, the present study extends the research on the mechanisms of organizational compassion on individual adversarial growth and provides a better understanding of job control as the boundary condition for the formation of individual adversarial growth.

### 5.2 Practical implications

Although the impact of the COVID-19 epidemic is waning, adversity is inevitable in the life course. Individuals can experience positive changes and obtain opportunities for growth as a result of adversity (Joseph, 2021). First, from an organizational perspective, a compassionate climate should be established and promoted within the organization, which should encourage managers and coworkers to provide emotional comfort and necessary assistance to employees who are experiencing adversity. Organizational compassion emphasizes that the organization as a whole “notices,” “feels,” and “responds” to the suffering of its members. Therefore, supervisors and organization members need to be empathic to others. Managers should try to listen to individuals in adversity and provide them with an outlet for their negative emotions. In addition, organizational resources and management policies should be used to help employees cope with adversity, e.g., flexible time arrangements, employee assistance programs, training, counseling services, low-interest or no-interest loans, legal support services, etc. Emotional and resource support provided by the organization and supervisors can be effective in helping employees recover from adversity, increase wellbeing, better engage in their work, and reduce the incidence of deviant behaviors in the workplace (Dodson and Heng, 2022; Paganin et al., 2023).

From an employee perspective, organizational compassion can be a powerful source of support and strength for individual recovery from adversity (Dodson and Heng, 2022). Everyone plays multiple roles in society, and each role is mutually supportive and interpenetrating. Individuals who are struggling in one role can find support not only within the role but also across roles. Work roles and life roles are considered to be the two most important social roles (Reiche et al., 2023). When faced with adversity at work, the individual can take advantage of the resources available in the organization and the emotional support of other organizational members; Individuals can also benefit from organizational compassion when they encounter difficulties in their personal lives. Job role fulfillment can help individuals gain confidence in coping with adversity and making positive changes. Therefore, individuals should actively seek the support and help of the organization when experiencing adversity.

Finally, organizational compassion has a more pronounced positive effect on employees with lower job control. Employees who have less autonomy over their work generally have a weaker sense of control, and adversity can further weaken their perceived control over their lives (Sawatsky et al., 2020). It can lead to a range of negative perceptions and behaviors. They have higher needs for care and support from external sources, and organizational compassion has a stronger impact on adversarial growth by increasing individual work passion and self-worth. Employees with higher job control are likely to derive satisfaction directly from their work, whereas employees with lower control are more dependent on emotional and policy support from the organization. Therefore, organizations should pay more attention to those employees with lower job control who are in adversity, they are more likely to benefit from the organization's care and compassion.

## 6 Research limitations and future research

There are some limitations in the study. First, the data in the study were all from Chinese companies, where the need for belonging is generally higher. Individuals are more dependent on the organization than in countries with individualistic cultures, so the study's findings may have some external validity issues. Future research could consider comparing the effects of organizational compassion on individual adversarial growth from different cultural contexts. Secondly, the self-report scales were used in this study, especially for adversity growth, which is still mainly evaluated from individual self-perceptions. Although the statistical analysis showed that the common method deviation was not serious in this study, it should still have some influence on the study findings. Subsequent studies may consider multiple methods such as experimental design and in-depth interviews to further validate the mechanisms that shape individual adversarial growth. Next, in this study, we did not make a distinction between harmonious work passion and obsessive work passion. The two types of passion for work may have different antecedents, and organizational compassion itself involves emotional, behavioral, and policy support for employees in adversity. It is possible that different types of compassion measures inspire different kinds of work passions, and a refined study on the impact of organizational compassion on adversarial growth through the two paths of harmonic work passion and forced work passion could be considered in future research. Finally, most of the respondents in this study are knowledge workers, whose intrinsic need for job autonomy is generally high. Meanwhile, they feel more attached to the organization and value their work role more. Therefore the perception in the organization has a greater impact on them than on other types of employees, the moderating effect of job control on the relations between organizational compassion, work passion, self-worth, and adversarial growth needs to be further verified.

Adversarial growth is gaining increasing attention in the post-epidemic era, and antecedents and consequences of adversarial growth deserve further exploration, especially from the organizational and work perspectives. In addition, the study of organizational compassion has been ongoing for some time, there is not yet a broad consensus on its structural dimensions and measurement. Future research may focus on the development of organizational compassion scales in different cultural contexts and under different industries, which could also give organizations clearer guidelines and help employees in adversity to benefit from it.

## Data availability statement

The data presented in this study are available on request from the corresponding author.

## Ethics statement

The studies involving humans were approved by the Research Ethics Committee of Business School in Macau University of Science and Technology. The studies were conducted in accordance with the local legislation and institutional requirements. The participants provided their written informed consent to participate in this study.

## Author contributions

TN: Conceptualization, Data curation, Formal analysis, Methodology, Resources, Software, Supervision, Writing – original draft, Writing – review & editing. XZ: Investigation, Software, Validation, Writing – review & editing. YZ: Investigation, Validation, Writing – original draft, Writing – review & editing.
